# AI-Based Prediction of Bone Conduction Thresholds Using Air Conduction Audiometry Data

**DOI:** 10.3390/jcm14186549

**Published:** 2025-09-17

**Authors:** Chul Young Yoon, Junhun Lee, Jiwon Kim, Sunghwa You, Chanbeom Kwak, Young Joon Seo

**Affiliations:** 1 Research Institute of Hearing Enhancement, Yonsei University Wonju College of Medicine, Wonju 26426, Republic of Korea; fezro@yonsei.ac.kr (C.Y.Y.); cksqja654@gmail.com (C.K.); 2 Department of Medical Informatics and Biostatistics, Yonsei University Wonju College of Medicine, Wonju 26426, Republic of Korea; 3 Department of Otorhinolaryngology, Yonsei University Wonju College of Medicine, 20 Ilsan-ro, Wonju 26426, Republic of Korea

**Keywords:** hearing-related disorders, bone conduction, air conduction, deep learning, digital phenotyping, big data

## Abstract

**Background/Objectives:** This study evaluated the feasibility of predicting bone conduction (BC) thresholds and classifying air–bone gap (ABG) status using only air conduction (AC) data obtained from pure tone audiometry (PTA). **Methods:** A total of 60,718 PTA records from five tertiary hospitals in the Republic of Korea were utilized. Input features included AC thresholds (0.25–8 kHz), age, and sex, while outputs were BC thresholds (0.25–4 kHz) and ABG classification based on 10 dB and 15 dB criteria. Five machine learning models—deep neural network (DNN), long short-term memory (LSTM), bidirectional LSTM (BiLSTM), random forest (RF), and extreme gradient boosting (XGB)—were trained using 5-fold cross-validation with Synthetic Minority Over-sampling Technique (SMOTE). Model performance was evaluated based on accuracy, sensitivity, precision, and F1 score under ±5 dB and ±10 dB thresholds for BC prediction. **Results:** LSTM and BiLSTM outperformed DNN in predicting BC thresholds, achieving ~60% accuracy within ±5 dB and ~80% within ±10 dB. For ABG classification, all models performed better with the 10 dB criterion than the 15 dB. Tree-based models (RF, XGB) achieved the highest classification accuracy (up to 0.512) and precision (up to 0.827). Confidence intervals for all metrics were within ±0.01, indicating stable results. **Conclusions:** AI models can accurately predict BC thresholds and ABG status using AC data alone. These findings support the integration of AI-driven tools into clinical audiology and telemedicine, particularly for remote screening and diagnosis. Future work should focus on clinical validation and implementation to expand accessibility in hearing care.

## 1. Introduction

Hearing loss (HL) is one of the most prevalent sensory impairments worldwide, affecting both quality of life and global health outcomes [1]. As of 2015, HL ranked as the fourth leading cause of years lived with disability (YLDs), accounting for 5.8% of all YLDs [2]. Globally, approximately 1.34 billion people—18.1% of the population—experience some degree of HL, with half a billion classified as having disabling HL [3,4]. The burden is particularly high in children and in low- and middle-income countries where access to early detection and intervention remains limited. Rapid and accurate hearing screening is therefore essential to support early diagnosis, timely treatment, and to mitigate the long-term personal and economic consequences of untreated HL.

Pure tone audiometry (PTA) is the gold standard for diagnosing HL. Since the 1920s, PTA has been widely used to assess the severity and type of HL by measuring hearing thresholds through two conduction pathways: air conduction (AC) and bone conduction (BC). AC assesses sound transmission through the outer, middle, and inner ear and is easier to administer, whereas BC bypasses the outer and middle ear, transmitting sound vibrations directly through the skull to the cochlea [5,6]. While AC thresholds are primarily used for quantifying HL severity and diagnosing sensorineural HL, BC is critical for identifying conductive HL by determining the presence of an air–bone gap (ABG). However, BC testing requires specialized equipment and is highly sensitive to examiner proficiency and oscillator placement, making it more difficult to perform—especially in pediatric or telemedicine settings.

Previous research has attempted to model and predict hearing thresholds or recovery using machine learning (ML) and deep learning (DL) techniques. Studies have applied regression models or neural networks to predict postoperative hearing, HL progression, or hearing aid outcomes [7,8,9,10,11]. However, no prior research has directly predicted BC thresholds using AC data. The fundamental differences in auditory transmission mechanisms between AC and BC have traditionally made this task challenging. Nonetheless, both methods ultimately stimulate the cochlea, implying potential correlations between their thresholds across frequencies.

Advances in artificial intelligence (AI)—particularly in DL—offer promising tools for identifying complex, nonlinear relationships in medical data. DL models such as neural networks have demonstrated strong performance in pattern recognition across various domains, including audiology. Leveraging large audiometric datasets, these models may be able to infer BC thresholds from AC measurements, potentially simplifying HL diagnosis by reducing reliance on cumbersome BC tests [9,12].

The aim of this study is to explore the feasibility of predicting BC thresholds using AC data from PTA through various DL models. If successful, this approach could improve the efficiency and accessibility of HL screening, particularly in telemedicine, mobile health environments, and resource-limited settings, by minimizing the need for direct BC measurements.

## 2. Materials and Methods

### 2.1. Data Collection and Participants

Data for this study were obtained from the Hearing Big Data Center (HBDC), which comprises PTA results from five tertiary hospitals in the Republic of Korea: Yonsei University Wonju Severance Christian Hospital, Gachon University Gil Hospital, The Catholic University of Korea Seoul St. Mary’s Hospital, Korea University Anam Hospital, and Hallym University Kangnam Sacred Heart Hospital. The dataset included patient age, sex, AC thresholds at frequencies ranging from 0.25 to 8 kHz, and BC thresholds at frequencies from 0.25 to 4 kHz. A total of 60,718 PTA records from 26,417 individuals were included after excluding entries with missing values across all AC and BC frequencies. In instances where AC or BC values were negative, they were replaced with 0. Null BC values were replaced with the corresponding AC value to allow modeling continuity.

This study received ethical approval from the Institutional Review Board of Yonsei University Wonju Severance Christian Hospital (CR324316). All data analyses were performed using Python v3.11.11 (Python Software Foundation, Wilmington, DE, USA), employing PyTorch v2.0.1+cu117 (Meta AI, Menlo Park, CA, USA) and Scikit-learn v1.6.1 (INRIA, Paris, France) for deep learning and machine learning implementations, respectively.

### 2.2. Experimental Design

**Figure 1:**
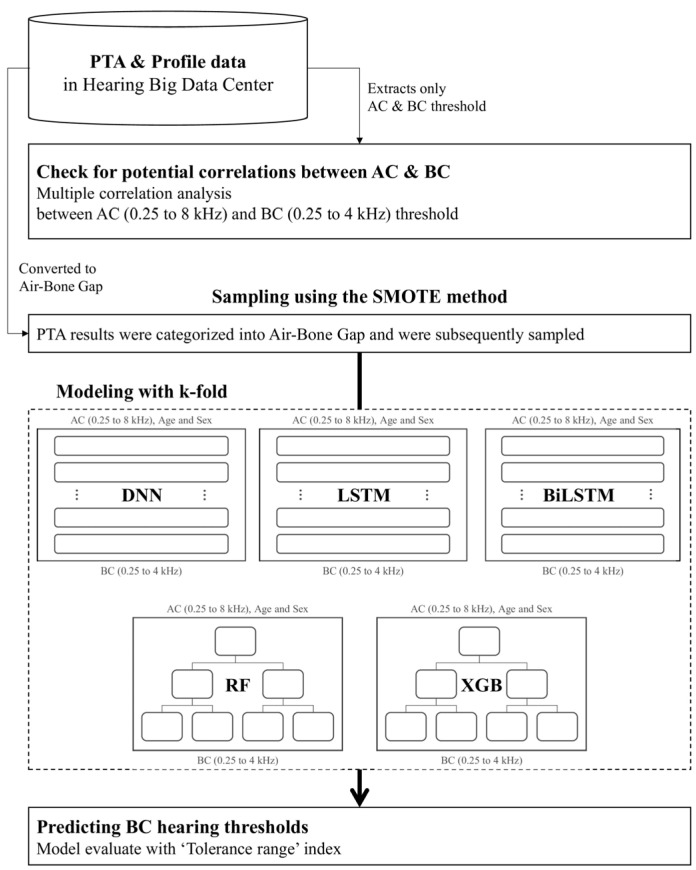
Flow chart of study.

Figure 1 illustrates the overall study workflow. After data preprocessing and sampling, models were trained to predict BC thresholds using AC thresholds, age, and sex as input features. In parallel, the presence of an ABG was classified based on differences between AC and BC thresholds. Five models were employed: Deep Neural Network (DNN), Long Short-Term Memory (LSTM), Bidirectional LSTM (BiLSTM), Random Forest (RF), and Extreme Gradient Boosting (XGB).

This study had two primary objectives: (1) to explore the correlation between AC and BC thresholds across frequencies and (2) to evaluate the accuracy of BC prediction using AC data. Additionally, the models were assessed for their ability to classify the presence of ABG. Accuracy for BC prediction was evaluated based on the “tolerance range,” which accounted for differences within ±5 dB and ±10 dB, reflecting the 5 dB step size used in clinical audiometry. For ABG classification, model performance was assessed using accuracy, sensitivity, precision, and F1 score derived from confusion matrices. ABG was defined as the difference between AC and BC thresholds equal to or exceeding 15 dB, and additionally at 10 dB to examine the possibility of BC underestimation.

### 2.3. Data Processing and Model Training

ABG classification was central to this study, and each PTA result was labeled accordingly. To address class imbalance, oversampling was performed using the Synthetic Minority Over-sampling Technique (SMOTE) [13]. The data processing of the study proceeded as shown in Table 1. The dataset was divided into training and test sets at a ratio of 8:2. The initial training dataset comprised 56,749 records, which increased to 64,000 after SMOTE was applied. The final dataset consisted of 70,937 records, with 14,188 reserved for testing.

**Figure 2:**
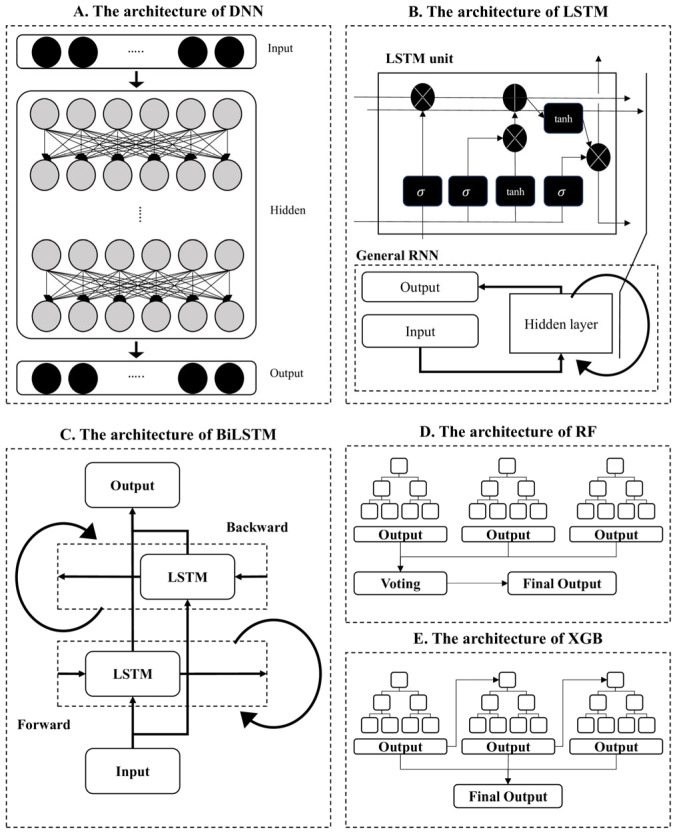
The architecture of each model. (**A**) The architecture of DNN; (**B**) the architecture of LSTM; (**C**) the architecture of BiLSTM; (**D**) the architecture of RF; (**E**) the architecture of XGB.

The deep learning models used in this study were DNN, LSTM, and BiLSTM. DNNs were constructed with multiple hidden layers (Figure 2A), while LSTM networks (Figure 2B) introduced memory units to capture temporal dependencies between features—critical for sequential data such as frequency-based hearing thresholds [14,15,16]. BiLSTM models (Figure 2C) enhanced this structure by processing information in both forward and backward directions, capturing bidirectional frequency relationships [17]. In contrast, RF (Figure 2D) is an ensemble model based on decision trees, using the bagging method to improve generalization and reduce overfitting [18]. XGB (Figure 2E), a gradient boosting framework, iteratively enhances prediction accuracy by focusing on the errors of previous trees [19]. DNN models are useful for capturing complex static patterns but lack sequential modeling ability. LSTM models are designed to process time-series or sequential data, making them suitable for auditory frequency prediction across bands. BiLSTM enhances this by capturing bidirectional patterns. We employed all three models to compare performance across architectures and to explore how directional and sequential learning influences BC threshold prediction.

All deep learning models were trained using the same configuration: 128 hidden units, mean squared error (MSE) as the loss function, ReLU activation, and the Adam optimizer. Training was performed for 500 epochs with a batch size of 32. For RF and XGB models, hyperparameters such as tree depth, minimum samples per leaf, and number of estimators were tuned through grid search optimization.

### 2.4. Evaluation

Model performance was validated using 5-fold cross-validation. Final evaluation was conducted on the independent test set, and all outcomes—including tolerance-based accuracy, ABG classification metrics, and confidence intervals—were reported as the mean values with 95% confidence intervals (CIs).

## 3. Results

### 3.1. Correlation Between AC and BC

**Figure 3:**
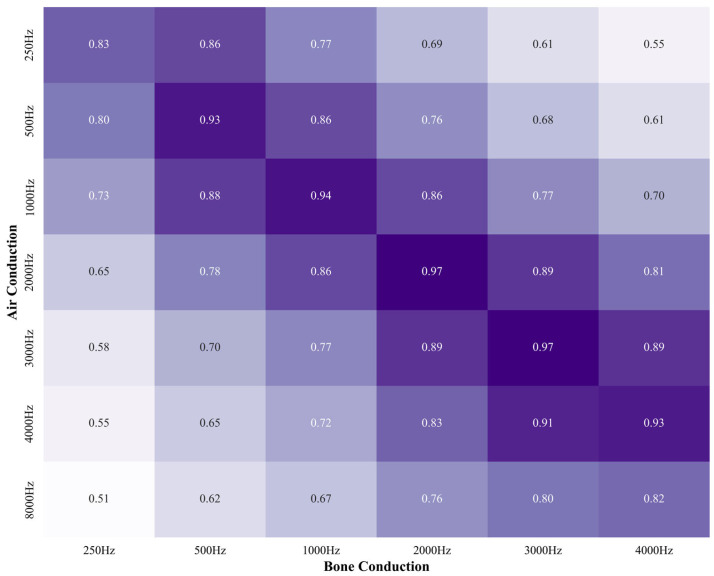
Correlation heatmap between air conduction and bone conduction hearing thresholds. Darker shades indicate stronger correlation coefficients in the heatmap.

To evaluate the feasibility of predicting BC from AC, a correlation analysis was conducted. AC and BC thresholds showed high correlations at corresponding frequencies, with Pearson coefficients of 0.83 (250 Hz), 0.93 (500 Hz), 0.94 (1 kHz), 0.97 (2 kHz), 0.97 (3 kHz), and 0.93 (4 kHz). AC at 8 kHz, despite lacking a corresponding BC frequency, showed moderate correlations (r = 0.76–0.82) with BC at 2–4 kHz. These findings indicate that AC is strongly correlated not only with matching BC frequencies but also with adjacent ones (Figure 3). Darker shades indicate stronger correlation coefficients in the heatmap.

### 3.2. BC Threshold Prediction from AC

**Figure 4:**
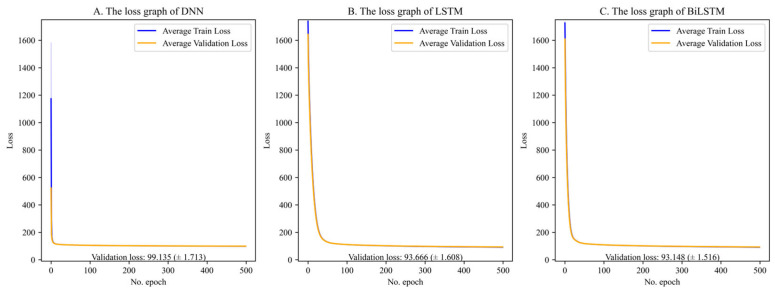
The loss graph of each model. (**A**) The loss graph of DNN; (**B**) the loss graph of LSTM; (**C**) the loss graph of BiLSTM.

Model training and validation were successfully performed (Figure 4). The final validation losses for the deep learning models were 99.135 (±1.713) for DNN (Figure 4A), 93.666 (±1.608) for LSTM (Figure 4B), and 93.148 (±1.516) for BiLSTM (Figure 4C), demonstrating stable training without divergence. Decision tree-based models (RF and XGB) do not use epoch-based learning; thus, optimization was conducted through hyperparameter tuning based on mean squared error.

Prediction accuracy was assessed based on whether the predicted BC thresholds fell within a clinical tolerance of ±5 dB or ±10 dB. The BiLSTM model achieved the highest accuracy among deep learning models: 0.643 (values) and 0.578 (patients) within ±5 dB, and 0.813 (values) and 0.838 (patients) within ±10 dB. LSTM followed closely, while DNN showed the lowest performance. Accuracy improved consistently with the wider ±10 dB range. The CIs for all accuracy metrics were below ±0.01, indicating stable predictions (Table 2).

Tree-based models outperformed deep learning models in BC prediction. RF achieved 0.724 (values) and 0.741 (patients) accuracy within ±5 dB, and 0.895 and 0.931 within ±10 dB. XGB yielded similar results, with 0.742 (values) and 0.714 (patients) within ±5 dB, and 0.892 and 0.933 within ±10 dB (Table 2).

### 3.3. ABG Classification

Predicted BC values were used to calculate ABG and classify patients under 10 dB and 15 dB criteria. Deep learning models performed better under the 10 dB threshold. BiLSTM achieved the highest F1 score (0.670), followed by LSTM (0.672) and DNN (0.658). At 15 dB, all deep learning models showed reduced performance, with identical accuracy (0.410) but slight differences in sensitivity and F1 scores. All CIs were within ±0.01 (Table 3). Interestingly, RF and XGB showed superior performance under the 15 dB criterion. Both models achieved 0.512 accuracy and approximately 0.545 F1 score at 15 dB but performed poorly at 10 dB, with accuracy dropping to 0.31 and F1 scores around 0.30. This suggests that while deep learning models are more suitable for detecting subtle ABGs (10 dB), tree-based models may be more reliable for broader thresholds (15 dB) (Table 3).

## 4. Discussion

PTA remains the global gold standard for hearing assessment due to its simplicity, non-invasiveness, and clinical utility in diagnosing both the degree and type of HL. PTA measures auditory thresholds through two pathways: AC, which assesses the entire auditory system from the outer ear to the cochlea, and BC, which bypasses the outer and middle ear to directly stimulate the cochlea via skull vibrations. While AC testing is relatively straightforward—requiring only headphones—BC testing is more cumbersome, relying on a bone oscillator, extended testing time, and examiner expertise [20]. Additionally, BC results are more susceptible to variability due to differences in oscillator placement and subject compliance, particularly in pediatric and telehealth contexts.

Despite these differences, both AC and BC ultimately assess cochlear response, suggesting a theoretical correlation between their thresholds. In clinical practice, BC is essential for calculating the ABG, a key indicator used to differentiate between conductive and sensorineural HL. Accurately predicting BC thresholds from AC data could significantly streamline the diagnostic workflow, reducing reliance on specialized equipment and examiner-dependent procedures. While the cost of bone vibrators is relatively low in many clinical settings, the benefit of AI-based BC prediction lies in its potential to improve accessibility in telemedicine and portable diagnostics, rather than direct cost replacement.

Our study confirmed a strong correlation between AC and BC thresholds, particularly at corresponding and adjacent frequencies. This supports the hypothesis that AC can be used to infer BC under certain conditions. Nevertheless, structural and functional differences between AC and BC transmission pathways introduce inherent challenges in establishing direct equivalence, particularly across variable clinical populations.

In this context, estimating the presence of an ABG—rather than the precise BC threshold—may represent a more feasible and clinically impactful application. ABG is a widely used clinical marker for identifying conductive components of HL, and predicting ABG using only AC thresholds could enable rapid HL classification in settings where BC measurement is not feasible. Our analysis demonstrated that ML and DL models can effectively support this task, offering new opportunities to optimize HL diagnostics.

We employed five AI models in this study—DNN, LSTM, BLSTM, RF, and XGB—to predict BC thresholds and ABG presence using AC data. Evaluation was structured around two main objectives: first, assessing the ability of models to predict BC values within defined clinical tolerance ranges (±5 dB and ±10 dB), and second, evaluating whether the predicted BC data could be used to classify patients as ABG-positive using both 10 dB and 15 dB criteria.

Among the deep learning models, BiLSTM consistently yielded the highest performance. All DL models achieved approximately 60% accuracy within a ±5 dB range and around 80% within ±10 dB, suggesting clinically acceptable accuracy in BC estimation. Performance improved progressively from DNN to LSTM and BiLSTM, reflecting the added benefit of modeling sequential relationships across frequency bands. While DNN models can effectively capture complex static patterns, they lack the ability to model temporal or sequential dependencies—an important limitation when dealing with frequency-based hearing data. RNN-based models, particularly BiLSTM, are better suited to this task, as they can interpret auditory frequency data in both forward and backward directions, effectively capturing frequency-to-frequency interactions.

Interestingly, RF and XGB—both tree-based models—outperformed DL models in overall accuracy for BC threshold prediction. These models excel in classification tasks due to their ensemble architecture, which constructs multiple decision trees to optimize predictive accuracy and reduce overfitting. RF achieved 72.4% accuracy for values and 74.1% for patients within ±5 dB, and 89.5% and 93.1%, respectively, within ±10 dB. XGB showed similar performance. However, while tree-based models provide excellent predictive accuracy, they lack the architectural capacity to model sequential or contextual relationships between adjacent frequencies. This may limit their effectiveness in tasks requiring continuous BC estimation across frequency ranges.

In ABG classification, model performance varied depending on the ABG threshold used. DL models performed better under the stricter 10 dB ABG criterion, whereas RF and XGB achieved better performance under the 15 dB threshold. At 10 dB, BiLSTM achieved the highest F1 score (0.670), with LSTM and DNN following closely. At 15 dB, RF and XGB outperformed DL models, each achieving 0.512 accuracy and approximately 0.545 F1 score. This suggests that while DL models are more adept at detecting subtle ABGs, tree-based models are more robust in identifying larger, more clinically established ABGs.

These findings highlight the complementary strengths of each modeling approach. DL models, particularly BiLSTM, are advantageous for modeling sequential patterns and predicting BC across frequencies, whereas tree-based models are effective for categorical classification tasks such as ABG detection. Combining these approaches provides a more comprehensive modeling strategy, allowing for flexibility depending on clinical context and diagnostic goals.

Model selection in this study was aligned with both the data characteristics and clinical objectives. DNNs were selected for their ability to capture non-linear, static patterns, while RNN-based models were incorporated to handle frequency-dependent sequential relationships. RF and XGB were included to assess the performance of ensemble-based decision algorithms, which have a proven track record in structured clinical data classification. This hybrid approach enabled us to evaluate model suitability across both regression (BC prediction) and classification (ABG detection) tasks.

Although the study demonstrated strong model performance, certain limitations must be acknowledged. The retrospective nature of the study limits control over the standardization of test environments, and PTA is inherently subjective compared to more objective measures like auditory brainstem response (ABR) or otoacoustic emissions (OAE). However, the data were collected from five tertiary hospitals in the Republic of Korea with high clinical standards, mitigating concerns about data quality. Additionally, the use of large-scale real-world data enhances the external validity of the findings.

In conclusion, this study presents a comprehensive evaluation of deep learning and machine learning models for predicting BC thresholds and ABG classification based on AC data. The results underscore the potential of AI-based tools to simplify hearing assessments, enhance screening accessibility, and support tele-audiology applications. Future research should explore prospective validation across diverse populations and further enhance model interpretability for clinical adoption.

One limitation of this study is its retrospective design, which relies on pre-existing data that may vary across institutions. The use of PTA as a reference standard introduces subjectivity influenced by patient cooperation and examiner technique. The model did not incorporate potentially relevant variables such as medical history or genetic factors, which could improve performance and generalizability. Additionally, data from Korean tertiary hospitals may limit applicability to other populations. Lastly, the interpretability of deep learning models remains a challenge for clinical adoption. Future research should address these issues through prospective validation and integration of multimodal, explainable approaches. Although overall correlations between AC and BC are strong, we observed that high-frequency AC thresholds are less predictive of low-frequency BC thresholds. This mismatch may limit clinical applicability in surgical contexts such as stapedotomy, where precise low-frequency BC information is critical for preoperative assessment. Therefore, AI-based predictions should be interpreted with caution, and not be solely relied upon for invasive treatment decisions without corroborating standard audiometric evaluations. Moving forward, incorporating additional clinical features—such as a patient’s otologic history, exposure to ototoxic drugs, or specific comorbidities—may enhance the prediction of BC thresholds and expand the clinical utility of AI-based audiometric models.

## 5. Conclusions

This study demonstrates that BC thresholds and ABG status can be feasibly predicted from AC data using artificial intelligence models. BiLSTM outperformed other deep learning models in BC estimation, while tree-based models like RF and XGB excelled in ABG classification. Prediction accuracy was higher at a ±10 dB tolerance and under the 10 dB ABG criterion. These findings support AI-assisted hearing assessment as a scalable alternative to direct BC testing, particularly in telemedicine or low-resource settings. Future studies should pursue external validation, integration of clinical variables, and improved model interpretability for real-world clinical application.

## Figures and Tables

**Table 1:** Result of data processing.

Air–Bone Gap	Raw	%	Train			
			Before SMOTE	%	After SMOTE	%
No	39,910	56.26	32,000	45.11	32,000	40.93
Yes	31,027	43.74	24,749	34.89	32,000	40.93
			Test			
			14,188	20.00	14,188	18.15
Total	70,937	100	70,937	100	78,188	100

SMOTE: Synthetic Minority Over-sampling Technique.

**Table 2:** Tolerance range by values and patients.

All Values	All Patients	Tolerance Range (±5 dB)		Tolerance Range (±10 dB)	
72,864	12,144	TRUE	Accuracy	TRUE	Accuracy
DNN	Values	53,648.6	0.632	67,977.8	0.799
	CI	(±153.64)	(±0.0)	(±137.99)	(±0.0)
	Patients	8065.6	0.568	11,556.0	0.814
	CI	(±23.39)	(±0.0)	(±29.55)	(±0.0)
LSTM	Values	54,486.8	0.641	69,052.2	0.811
	CI	(±774.23)	(±0.01)	(±498.84)	(±0.01)
	Patients	8114.0	0.572	11,882.8	0.838
	CI	(±215.32)	(±0.01)	(±97.03)	(±0.01)
BiLSTM	Values	54,738.4	0.643	69,212.8	0.813
	CI	(±261.44)	(±0.0)	(±169.33)	(±0.0)
	Patients	8201.8	0.578	11,884.4	0.838
	CI	(±72.69)	(±0.01)	(±34.96)	(±0.0)
RF	Values	53,949.7	0.741	64,698.4	0.895
	CI	(±27.76)	(±0.0)	(±16.9)	(±0.0)
	Patients	8769.8	0.724	11,332.2	0.931
	CI	(±10.26)	(±0.0)	(±3.09)	(±0.0)
XGB	Values	54,168.8	0.742	64,537.8	0.892
	CI	(±38.55)	(±0.0)	(±22.25)	(±0.0)
	Patients	8649.0	0.714	11,337.5	0.933
	CI	(±14.41)	(±0)	(±4.43)	(±0)

DNN: Deep Neural Network, LSTM: Long Short-Term Memory, BiLSTM: Bidirectional Long Short-Term Memory, RF: Random Forest, XGB: Extreme Gradient Boosting, CI: confidence interval. Values: Accuracy for each threshold value, Patients: Accuracy for patient-level predictions.

**Table 3:** Confusion matrix and evaluation indicators for air–bone gap prediction.

Model	Gap	Accuracy	Sensitivity	Precision	F1
DNN	15 dB	0.41	0.338	0.785	0.473
	CI	(±0.0)	(±0.0)	(±0.01)	(±0.0)
	10 dB	0.586	0.542	0.837	0.658
	CI	(±0.0)	(±0.01)	(±0.0)	(±0.0)
LSTM	15 dB	0.41	0.355	0.778	0.487
	CI	(±0.0)	(±0.01)	(±0.01)	(±0.01)
	10 dB	0.586	0.566	0.827	0.672
	CI	(±0.0)	(±0.01)	(±0.0)	(±0.01)
BiLSTM	15 dB	0.41	0.353	0.78	0.486
	CI	(±0.0)	(±0.0)	(±0.0)	(±0.0)
	10 dB	0.586	0.562	0.83	0.67
	CI	(±0.0)	(±0.01)	(±0.0)	(±0.01)
RF	15 dB	0.512	0.408	0.826	0.546
	CI	(±0.0)	(±0.0)	(±0.0)	(±0.0)
	10 dB	0.31	0.192	0.739	0.305
	CI	(±0.0)	(±0.0)	(±0.0)	(±0.0)
XGB	15 dB	0.512	0.405	0.827	0.544
	CI	(±0.0)	(±0.0)	(±0.0)	(±0.0)
	10 dB	0.31	0.188	0.739	0.3
	CI	(±0.0)	(±0.0)	(±0.0)	(±0.0)

DNN: Deep Neural Network, LSTM: Long Short-Term Memory, BiLSTM: Bidirectional Long Short-Term Memory, RF: Random Forest, XGB: Extreme Gradient Boosting, CI: confidence interval. Values: Accuracy for each threshold value, Patients: Accuracy for patient-level predictions.

## Data Availability

The audiometric datasets used in this study were obtained from the Hearing Big Data Center (HBDC) of five tertiary hospitals in the Republic of Korea. Data are not publicly available due to patient privacy protection and institutional review board (IRB) restrictions, but may be available from the corresponding author upon reasonable request and with permission from the respective institutions.

## Abbreviations

The following abbreviations are used in this manuscript:

HL	Hearing loss
YLDs	Years lived with disability
PTA	Pure tone audiometry
AC	Air conduction
BC	Bone conduction
ABG	Air–bone gap
ML	Machine learning
DL	Deep learning
AI	Artificial intelligence
HBDC	Hearing Big Data Center
DNN	Deep Neural Network
LSTM	Long Short-Term Memory
BiLSTM	Bidirectional Long Short-Term Memory
RF	Random Forest
XGB	Extreme Gradient Boosting
SMOTE	Synthetic Minority Over-sampling Technique
MSE	Mean squared error
CIs	Confidence intervals
ABR	Auditory brainstem response
OAE	Otoacoustic emissions
